# Cleavage and Structural Transitions during Maturation of *Staphylococcus aureus* Bacteriophage 80α and SaPI1 Capsids

**DOI:** 10.3390/v9120384

**Published:** 2017-12-16

**Authors:** James L. Kizziah, Keith A. Manning, Altaira D. Dearborn, Erin A. Wall, Laura Klenow, Rosanne L. L. Hill, Michael S. Spilman, Scott M. Stagg, Gail E. Christie, Terje Dokland

**Affiliations:** 1 Department of Microbiology, University of Alabama at Birmingham, Birmingham, AL 35294, USA; kizziah4@uab.edu (J.L.K.); k1797732@uab.edu (K.A.M.); rozythorn@gmail.com (R.L.L.H.); 2 Protein Expression Laboratory, National Institute of Arthritis and Musculoskeletal and Skin Diseases, The National Institutes of Health, Bethesda, MD 20892, USA; altaira.dearborn@nih.gov; 3 Department of Microbiology and Immunology, Virginia Commonwealth University, Richmond, VA 23298, USA; erin.wall@nih.gov (E.A.W.); laura.klenow@fda.hhs.gov (L.K.); gail.christie@vcuhealth.org (G.E.C.); 4 Institute of Molecular Biophysics, Florida State University, Tallahassee, FL 32306, USA; mspilman@directelectron.com (M.S.S.); sstagg@fsu.edu (S.M.S.)

**Keywords:** Caudovirales, virus assembly, capsid size determination, cryo-electron microscopy, *Staphylococcus aureus* pathogenicity islands, SaPI1

## Abstract

In the tailed bacteriophages, DNA is packaged into spherical procapsids, leading to expansion into angular, thin-walled mature capsids. In many cases, this maturation is accompanied by cleavage of the major capsid protein (CP) and other capsid-associated proteins, including the scaffolding protein (SP) that serves as a chaperone for the assembly process. *Staphylococcus aureus* bacteriophage 80α is capable of high frequency mobilization of mobile genetic elements called *S. aureus* pathogenicity islands (SaPIs), such as SaPI1. SaPI1 redirects the assembly pathway of 80α to form capsids that are smaller than those normally made by the phage alone. Both CP and SP of 80α are N-terminally processed by a host-encoded protease, Prp. We have analyzed phage mutants that express pre-cleaved or uncleavable versions of CP or SP, and show that the N-terminal sequence in SP is absolutely required for assembly, but does not need to be cleaved in order to produce viable capsids. Mutants with pre-cleaved or uncleavable CP display normal viability. We have used cryo-EM to solve the structures of mature capsids from an 80α mutant expressing uncleavable CP, and from wildtype SaPI1. Comparisons with structures of 80α and SaPI1 procapsids show that capsid maturation involves major conformational changes in CP, consistent with a release of the CP N-arm by SP. The hexamers reorganize during maturation to accommodate the different environments in the 80α and SaPI1 capsids.

## 1. Introduction

The Caudovirales represent the most common group of bacteriophages (phages) with double-stranded DNA genomes. These phages share many structural characteristics, including an icosahedral or prolate capsid (or head) filled with DNA, and a tail attached to one vertex of the capsid via a portal (or connector), through which the DNA is ejected during an infection. The capsids are assembled from a major capsid protein (CP) as empty precursor procapsids into which the DNA is packaged through the portal by the phage terminase. In most phages, procapsid assembly also requires a separate scaffolding protein (SP) that functions as an assembly chaperone. (One group, the HK97-like phages, does not encode a separate SP, but instead has a scaffolding domain fused to the CP.) During packaging, the capsids undergo structural transformations, including an expansion and a transition from a rounded to a more angular shape, expelling the SP in the process. In many bacteriophages, maturation is preceded or accompanied by cleavage of CP and/or SP. Tails are attached to the mature capsids after DNA packaging.

*Staphylococcus aureus* is an opportunistic human pathogen often associated with serious infections, especially of skin and soft tissues [1,2]. The emergence of virulent, antibiotic-resistant *S. aureus* strains has become a significant public health problem [3,4]. Most antibiotic resistance and virulence genes in *S. aureus* are carried on mobile genetic elements (MGEs) [5,6]. Since most *S. aureus* strains are not naturally transformable, these MGEs are generally transferred via transduction by bacteriophages [7].

**Figure 1:**
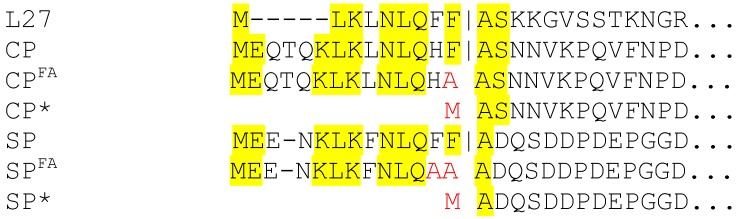
N-terminal sequences of ribosomal protein L27, 80α CP and 80α SP, showing the Prp cleavage site (vertical line). The sequences of the precleaved (CP* and SP*) and uncleavable (CP^FA^ and SP^FA^) proteins are also shown. Completely conserved residues in all sequences are indicated in yellow. Mutated residues are shown in red letters.

80α is a typical example of a staphylococcal transducing phage, capable of transferring non-specific chromosomal or plasmid DNA at low frequency [8,9]. The 80α virion has a 63 nm icosahedral head with *T* = 7 architecture, connected to a 190 nm long flexuous tail, capped by an elaborate baseplate [10]. 80α procapsids are formed from 415 copies of CP, 130–320 copies of SP, a dodecameric portal protein, and a few copies of a minor capsid protein (gp44) of unknown function [11]. We previously demonstrated that both CP and SP are processed by a host protease that we designated Prp [12]. Prp cleaves between F and A, removing 13 and 14 residues from the N-termini of CP and SP, respectively (Figure 1). Prp is also involved in cleavage of ribosomal protein L27 during ribosome assembly, and is essential for cell function [13]. L27, CP and SP all share a conserved N-terminal sequence at the Prp cleavage site (Figure 1). DNA packaging occurs by a headful mechanism, requiring the action of the terminase complex, consisting of a large (TerL) and a small (TerS) subunit. Packaging is concurrent with loss of SP and capsid expansion.

In addition to generalized transduction, 80α and many related staphylococcal phages also serve as “helper” phages for the high frequency mobilization of *S. aureus* pathogenicity islands (SaPIs), ≈15 kb MGEs that carry genes encoding virulence factors, especially superantigen toxins [14]. The SaPIs are normally stably integrated into their host genomes through expression of a master repressor, Stl, but are mobilized by the helpers upon interaction of Stl with early lytic phage proteins that act as derepressors [15]. The various SaPIs have highly divergent Stl proteins that respond to different phage-encoded derepressors. Phage 80α is able to mobilize several SaPIs, including SaPI1, SaPI2, SaPIbov1 and SaPIbov2 [15,16]. Conversely, some SaPIs, such as SaPIbov1, can respond to more than one phage derepressor, thereby maximizing their survival upon infection with a range of phages [17,18,19]. After excision and replication, the SaPI genomes are packaged into transducing particles made from phage-encoded structural proteins. Many SaPIs, such as SaPI1, redirect the 80α capsid assembly pathway to form capsids that are smaller than the normal phage capsids and that are thus incapable of packaging full-length phage genomes [15]. This size redirection is dependent on the SaPI-encoded CpmA and CpmB proteins [20,21]. CpmB is an α-helical dimer that acts as a scaffolding protein and competes with the 80α SP for a binding site on CP [22]. Both SP and CpmB are expelled from the capsid during packaging and maturation, presumably through pores in the procapsid that close upon maturation. The role of CpmA is less clear, but might be involved in providing access to CP for CpmB by removing the SP. SaPI1 also encodes a TerS subunit that recognizes a specific *pac* site in the SaPI1 genome [23], ensuring preferential packaging of SaPI1 genomes into the resulting transducing particles.

In this study, we have investigated the 80α and SaPI1 capsid maturation and the role of the Prp-mediated cleavage in this process. We show that assembly and maturation of 80α procapsids is independent of CP cleavage, but that capsid formation requires the presence of an intact SP N-terminal sequence. We recently presented reconstructions of 80α and SaPI1 procapsids at 3.8 Å resolution [22], and have previously reported the low-resolution structure of the 80α mature capsid [10]. Here, we report the structures of a mature 80α capsid made from an uncleavable version of CP at 5.2 Å resolution and of a wildtype SaPI1 mature capsid at 8.4 Å resolution. These structures reveal the architectural reorganization that occurs upon capsid maturation and suggest how maturation is coupled to SP release. Comparisons of the 80α and SaPI1 capsids show how the hexamers reorganize to accommodate the difference in curvature and environment in the small and large capsids.

## 2. Materials and Methods

### 2.1. Allelic Exchange

Allelic exchange was carried out using the pMAD exchange vector [24], as previously described [11,22]. The desired mutant alleles for CP and SP were introduced by PCR into fragments that were inserted into BamHI/NcoI-cleaved pMAD using the Clontech In-Fusion HD cloning kit. The resulting pMAD derivatives and the primers used for generating them are listed in Tables S1 and S2. After transformation of *E. coli* Stellar^TM^ cells (Clontech, Mountain View, CA, USA) with the resulting plasmids and confirmation by DNA sequencing, *S. aureus* strain RN4220 or the RN4220-derived 80α lysogen RN10616 were transformed with these plasmids by electroporation. For ST208 and ST209, the plasmids were transferred from RN4220 into the RN450-derived 80α ΔCP lysogen JP3569 by transduction with 80α. The transformed strains were plated on tryptic soy agar (TSA) with 5 µg/mL erythromycin and 200 µg/mL X-Gal at 42 °C. Blue colonies were subjected to repeated cycles of growth at 30 °C and 42 °C without erythromycin to cure the cells of the plasmid. White colonies were screened by PCR and sequencing to confirm the desired mutation.

### 2.2. Preparation and Titering of Phage Samples

Lysogenic *S. aureus* strains (Table 1) were grown at 32 °C in 25:1 CY media with β-glycerophosphate [25], induced with mitomycin C (1 µg/mL) and purified by precipitation with polyethylene glycol (PEG) 6000 and CsCl gradient centrifugation, as previously described [11,22]. The purified phage was serially diluted and plated on *S. aureus* RN4220 to determine phage titers.

### 2.3. Phage Stability Assays

Phage stability was assessed by inducing DNA ejection at 63 °C in phage buffer (20 mM Tris pH 8.0, 100 mM NaCl, 1 mM MgSO_4_, 4 mM CaCl_2_) for up to 40 min, followed by degradation of the ejected DNA with 50 µg/mL DNase I for 1 h at 37 °C. The reaction was stopped by the addition of 10 mM EDTA, followed by disruption of the capsids with 1 mg/mL proteinase K for 1 h at 55 °C. The released DNA was then separated by electrophoresis on a 0.7% agarose gel in Tris/Borate/EDTA (TBE) buffer.

### 2.4. Electron Microscopy

Samples for electron microscopy (EM) were dialyzed into phage dialysis buffer (10 mM Tris-HCl pH 8.0, 50 mM NaCl, 1 mM MgSO_4_, 4 mM CaCl_2_). For negative stain, 3 µl of sample was applied to glow-discharged continuous carbon film on 400 mesh Cu grids and stained with 1% uranyl acetate. For cryo-EM, the dialyzed samples were applied to glow-discharged Quantifoil R2/1 holey film on Cu grids and vitrified by plunging into liquid ethane using an FEI Vitrobot Mark IV (5 s blot time, blot pressure 5). The SaPI1 virions were imaged in an FEI Tecnai F20 electron microscope operated at 200 kV, and the data was collected on SO-163 film at a magnification of 62,000× with defocus levels from 1.0–2.5 µm and electron dose of ≈25 e^−^/Å^2^. The images were digitized at 4000 dpi on a Nikon 9000ED film scanner and binned to 2000 dpi, corresponding to 2.05 Å/pixel. The 80α CP^FA^ virions (strain ST208) were imaged using Leginon [30] on an FEI Titan Krios operated at 120 kV. Data was collected on a Gatan Ultrascan 4000 CCD detector at a magnification of 96,000×, corresponding to 0.92 Å/pixel. 5624 images were collected with a nominal defocus range 1.25–3 µm and a total electron dose of 15 e^−^/Å^2^.

### 2.5. Three-Dimensional Reconstruction and Model Building

The 80α CP^FA^ data was initially processed using Appion [31]. Particles were picked using DoGPicker [32]. A template was made and used to pick 17,946 particles by template matching in FindEM [33]. The contrast transfer functions (CTF) for all micrographs were estimated with ACE [34]. Images with poorly estimated CTF were discarded, resulting in a stack of 12,503 particles. The particle images were then reconstructed using the program *jspr*, essentially following the procedure in Guo and Jiang [35]. The final reconstruction, using 11,843 particles, reached a resolution of 5.2 Å by the FSC_0.143_ criterion. A molecular model was built into the ST208 reconstruction using the CP model from the previously determined procapsid reconstruction [22] as a starting point. Model building and real-space refinement were done using Coot [36], followed by refinement with REFMAC5 [36] as an all-alanine polypeptide, due to the scarcity of clear side chain density in the map at this resolution.

The SaPI1 virion images from the scanned films were processed in EMAN 1.9 [37], resulting in 6471 particle images, and reconstructed using AUTO3DEM [38]. A starting model was generated by assigning random starting orientations to several data subsets, about half of which converged to a higher resolution structure with the expected *T* = 4 symmetry. The final resolution for the SaPI1 reconstruction was 8.4 Å according to the FSC_0.143_ criterion. Subunits A–D from the 80α CP^FA^ subunits were rigid body fitted into the SaPI1 density, and subjected to real-space refinement in Coot, followed by one cycle of REFMAC5 refinement.

All maps were sharpened with an empirically determined inverse B factor using the program *bfilter* from the *bsoft* suite [39]. Map display and density operations were done in UCSF Chimera [40].

## 3. Results

### 3.1. Cleavage of Capsid and Scaffolding Protein

To assess the role of cleavage of CP and SP by Prp in the 80α assembly process, we generated mutant phages that produced the pre-cleaved version of CP (CP*; strain ST209) or SP (SP*; strain ST247), or versions in which the F|A cleavage site had been mutated to A|A (FF|A to AA|A in SP), resulting in uncleavable CP (CP^FA^; strain ST208) or SP (SP^FA^; strain ST379) (Table 1; Figure 1).

**Figure 2:**
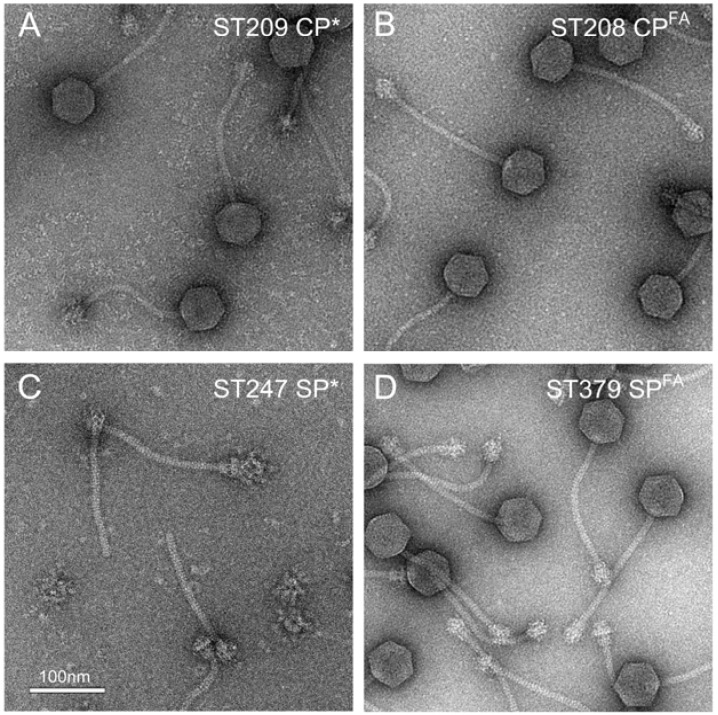
Electron micrographs of negatively stained assembly products formed by 80α mutants: (**A**) 80α CP* (pre-cleaved CP; strain ST209); (**B**) 80α CP^FA^ (uncleavable CP; strain ST208); (**C**) 80α SP* (pre-cleaved SP; Strain ST247); (**D**) 80α SP^FA^ (uncleavable SP; strain ST379). Scale bar = 100 nm.

Except for the pre-cleaved SP mutant ST247, all mutants produced DNA-filled phage particles that appeared to be identical to the wildtype and had normal viability (Figure 2; Table 2). SDS-PAGE confirmed that CP^FA^ virions contained mostly uncleaved CP, although some cleaved protein was also present (Figure S1). ST247 produced no viable phage, exhibited greatly reduced CP production, and accumulated large numbers of tails. This phenotype was very similar to strains in which the SP gene had been deleted (ST91) [29], indicating that the N-terminal sequence of SP was absolutely essential for assembly and viability.

**Figure 3:**
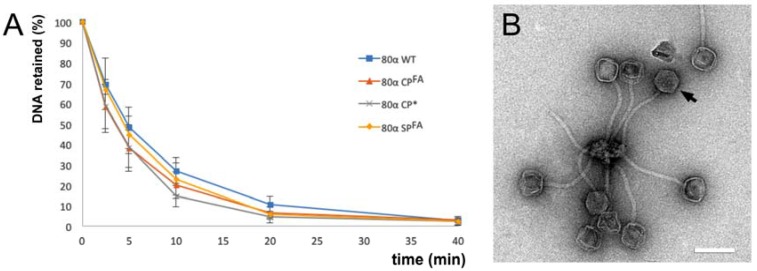
DNA stability of mutants. (**A**) The curves show the fraction of DNA remaining in the capsids as a function of time of incubation at 63 °C, measured by intensity of bands on an agarose gel, for 80α WT (blue), 80α CP^FA^ (red), 80α CP* (gray) and 80α SP^FA^ (orange). Average and standard deviation of three measurements are shown. Intensities were normalized to those of each mutant at time zero. (**B**) Electron micrograph of 80α WT after 40 min at 63 °C, showing ejection of DNA without disruption of the capsids themselves. One full capsid protected from ejection by baseplate clustering is indicated (arrow). Scale bar = 100 nm.

We had previously observed that 80α WT would eject its DNA over the course of hours to days at room temperature. To test whether the mutant phages exhibited altered DNA stability, they were subjected to a DNA stability assay, with 80α WT as a control. (ST247, which produced no capsids, was not included.) In this assay, the phages were incubated at 63 °C for up to 40 min. Samples were taken at specific time points and treated with DNase I to remove ejected DNA, and the remaining DNA was separated by agarose gel electrophoresis. All capsids released their DNA almost completely after 40 min, and the rate of release was similar to the WT for all mutants (Figure 3A). The end points of the reactions were subjected to negative stain EM, which showed intact, but empty particles, demonstrating the capsids themselves were not disrupted by the heat treatment (Figure 3B). The baseplates tend to cluster together after heat treatment, probably due to exposure of hydrophobic sequences, thereby preventing DNA ejection from a few remaining phage particles.

### 3.2. Structure of the Mature 80α Capsid

We recently determined structures of 80α and SaPI1 procapsids to 3.8 Å and 3.7 Å, respectively, from data collected at Florida State University (FSU) on an FEI Titan Krios equipped with a DE-20 direct electron detector [22]. These structures allowed most of the CP and portions of SP and CpmB to be modeled into the density, and suggested that CpmB acts as an alternative internal scaffold that changes the angles between capsomers, resulting in the smaller *T* = 4 shell. We also previously determined the structure of the 80α mature capsid at ≈10Å resolution from data collected on film, which showed that maturation involved a straightening of the spine helix (α3) and minor changes in angle between A and P domains [10]. However, this structure was at insufficient resolution to allow the complete backbone to be built into the density with confidence, and the E-loop, P-loop and N-arm were not resolved in the maps.

**Figure 4:**
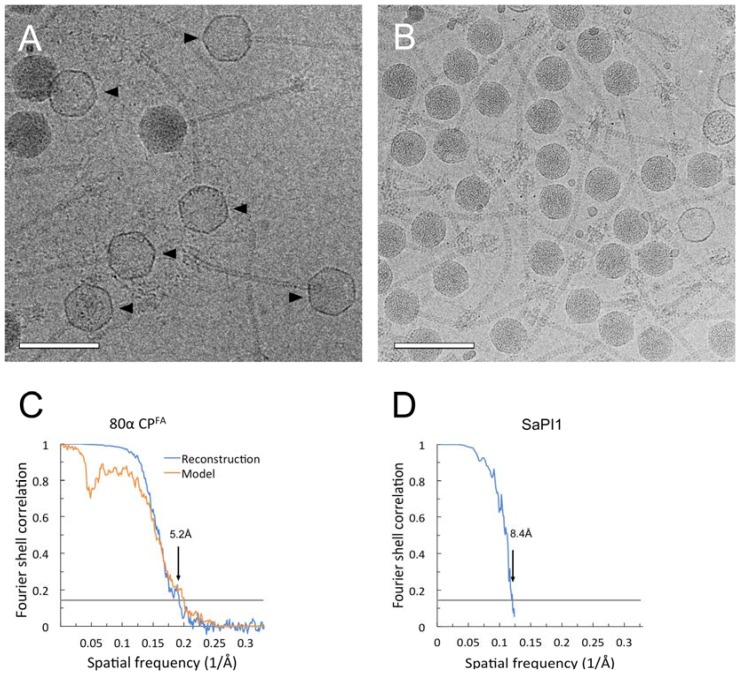
Cryo-electron micrographs of 80α CP^FA^ (**A**) and SaPI1 (**B**) virions. Empty capsids are indicated by arrowheads in A. Scale bar = 100 nm. Fourier Shell Correlation (FSC) plots between the two half datasets (blue) for the 80α CP^FA^ (**C**) and SaPI1 (**D**) reconstructions. The resolution at FSC = 0.143 is indicated. Correlation between the model and the map is shown for 80α CP^FA^ (orange), but not for the low-resolution SaPI1 reconstruction. (The curve in D was generated by AUTO3DEM, which only calculates FSC up to the resolution used for map generation, in this case 8Å).

Here, CP^FA^ virions were imaged at FSU using the Titan Krios microscope, but prior to the installation of the direct electron detector, and the images were therefore collected with a CCD camera. Reasoning that empty particles would be more amenable to 3D reconstruction, since the lack of internal DNA would reduce the background noise, we chose a sample in which the majority of the capsids had ejected their DNA upon prolonged exposure at 22 °C (Figure 4A). A total of 11,843 empty capsid images were picked from the CCD frames and subjected to icosahedral reconstruction using the program *jspr* [35]. The reconstruction reached a final resolution of 5.2 Å by the FSC_0.143_ criterion (Figure 4C).

**Figure 5:**
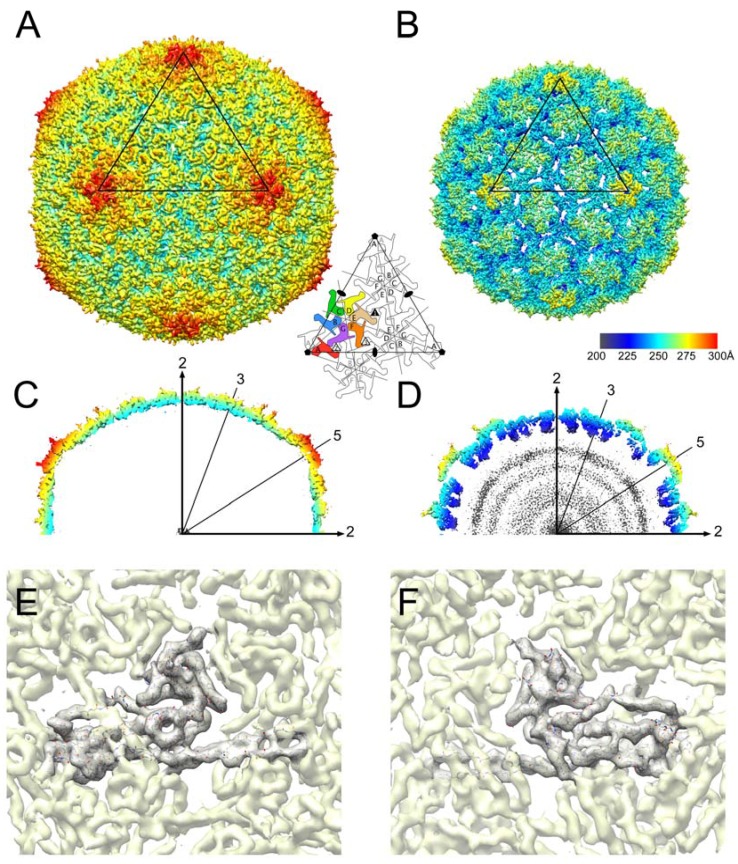
Isosurface representations of the reconstructions of the mature 80α CP^FA^ (strain ST208) capsid (**A**) and the previously determined 80α procapsid (**B**) [22], colored radially by distance from the center of the capsid according to the color bar (Å). One triangular face, delimited by three fivefold axes of symmetry, is indicated on the isosurface, and corresponds to the schematic diagram that shows the *T* = 7 architecture with seven CP subunits in the asymmetric unit. (**C**,**D**) Central sections of the 80α CP^FA^ mature capsid (**C**) and the procapsid (**D**), colored as in A and B. Symmetry axes are indicated. (**E**,**F**) Details of the density for the 80α CP^FA^ mature capsid viewed from the outside (**E**) and inside (**F**) of the capsid shell. The density corresponding to CP subunit C is shown as a mesh, while the surrounding density is shown as a semi-transparent yellow surface. The atomic model for CP subunit C (as a poly-Ala model) is shown in the density.

The resulting reconstruction shows the previously described *T* = 7 architecture with seven CP subunits (denoted A–G) in the asymmetric unit, organized into A_5_ hexamers and BCDEFG pentamers on an icosahedrally shaped capsid (Figure 5A). Compared to the procapsid (Figure 5B), the mature capsid is larger and more angular and has a thinner shell (Figure 5C,D). While most side chains could not be resolved in the map at this resolution, the backbone could be clearly traced through the map (Figure 5E,F), and was modeled using the previously determined procapsid structure as a starting point. After initial adjustment of the P- and A-domains, regions that did not fit in the density were manually re-built in O and Coot, followed by refinement in Coot and REFMAC. Side chains beyond the Cβ atom were omitted during refinement because of the low resolution of the map (Figure 5E,F). No density could be assigned for the N-terminal 30 residues of CP, including the N-terminal 14 residues that are normally cleaved from WT capsids. As expected, there was no density corresponding to the SP in the mature capsid.

**Figure 6:**
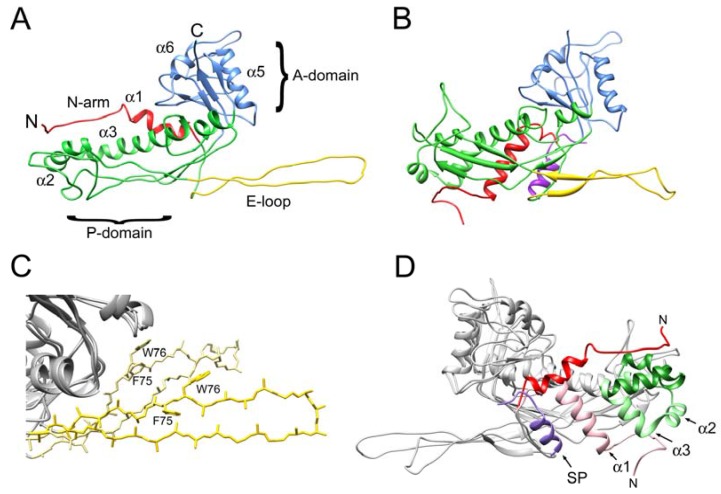
Comparison of 80α procapsid and mature capsid. (**A**,**B**) Ribbon diagrams of capsid protein subunit C from the 80α CP^FA^ mature capsid (**A**) and the procapsid (**B**), colored according to structural features: N-arm, red; E-loop, yellow; P-domain, green; A-domain, blue. SP in the procapsid, purple. (**C**) Superposition of the E-loops from subunit C in the 80α mature capsid (dark yellow) and procapsid (light yellow). Only the backbones and Cβ atoms are shown, except for residues F75 and W76 (indicated). (**D**) Superposition of CP subunit C from the 80α CP^FA^ mature capsid and procapsid viewed from the inside of the capsid. The N-arm (red) and spine helix (green) in the mature capsid are highlighted and compared to the same in the procapsid, colored pink and light green, respectively. SP (purple), α1, α2, α3 and the N-terminus are indicated on the procapsid model.

There are considerable differences between the CP subunit in the mature capsid compared to the procapsid, especially in the N-arm, E-loop, P-loop and part of the spine helix (α3) (Figure 6A,B). The E loop has a more flattened and extended conformation in the mature capsid, leading to a relaxation of the geometry for residues F75 and W76 (Figure 6C). The most striking difference in the CP subunit between procapsid and mature capsid is in the N-arm (residues 31–61; red and pink in Figure 6D). In the procapsid, the N-arm consists of a β-strand that adds to the β-sheet underneath the P-domain, followed by an α-helix (α1) that folds against the P-domain on the inside of the shell and interacts with the C-terminal α-helix of SP (purple in Figure 6D). Together, the SP, the N-arm and the spine helix form a “palisade” of α-helices. In the mature capsid (which has no SP), the β-strand folds underneath the E-loop that extends from the adjacent subunits, while α1 is rotated by 81° towards the outside of the capsid relative to its orientation in the procapsid (Figure 6D). This is accompanied by a straightening of the spine helix (α3) and major changes in the preceding α2 helix and the P-loop (green in Figure 6D), presenting a concerted rotation of the subunit that leads to a disruption of the α-helical palisade seen in the procapsid (Supplementary Movie 1).

The N-arm was not apparent in the previously described reconstruction of the 80α WT mature capsid [10]. We considered the possibility that the lack of CP cleavage in ST208 had locked down the N-terminus of CP, leading to the ordered conformation of the N-arm observed here. We went back to the original 80α WT film data and reprocessed it with AUTO3DEM. However, the resolution of the map did not improve beyond 10 Å. However, when the 80α CP^FA^ model was placed in a sharpened version of this map, density corresponding the N-arm could be seen when the density cutoff level was lowered (Figure S2). Furthermore, the N-arm was visible in the SaPI1 reconstruction, which also contained normally processed CP (see Section 3.3, below). Thus, it is most likely that the improved resolution of the map itself led to the better-resolved N-arm in the 80α CP^FA^ map.

**Figure 7:**
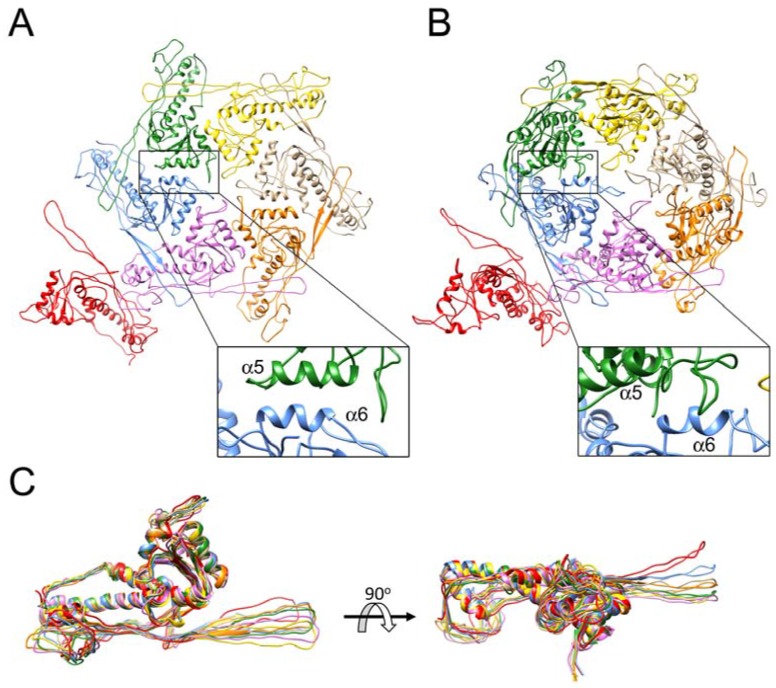
Ribbon diagrams showing one asymmetric unit from the 80α CP^FA^ mature capsid (**A**) and procapsid (**B**). The subunits are colored as in the schematic diagram in Figure 5 and according to Dearborn et al. [22]. The insets show close-up views of the interactions between subunit B α6 (blue) and subunit C α5 (green). (**C**) Superposition of all seven subunits (A–G) from the 80α CP^FA^ mature capsid, colored as in A. The right panel is rotated by 90° relative to the left panel.

During maturation, the CP subunits are rotated from a perpendicular orientation in which the A domains protrude on the surface of the capsid, to a lateral orientation in which both A- and P-domains lie tangentially in the plane of the shell. This rotation is accompanied by a reorganization of the asymmetric, skewed hexamers to almost perfectly six-fold symmetric hexamers (Figure 7A,B). Such reorganization is consistent with a change in location of the hexamers from the spherical surface of the procapsid to the flat face of the icosahedral mature capsid, since perfect hexagons cannot be fitted onto a sphere without leaving gaps between them. The pentamers, which sit on fivefold axes of symmetry, are by necessity symmetrical in both capsids, because of the imposed icosahedral symmetry. In both procapsids and mature capsids, the density was weaker and the local resolution was worse for the pentamers, consistent with some degree of disorder, perhaps resulting from the crowding of the A-domains [22]. (A weakening of the density by ≈8%, or 1/12, would be expected in both procapsids and mature capsids due to averaging with the unique portal vertex.) The symmetrization is accompanied by a regularization of the interactions between A domains, and the α5 and α6 helices form a tighter connection in the mature capsid (Figure 7A,B).

In the procapsids, the hexamer skew is accommodated by distinct differences in interactions between the A-domains and by conformational changes in the E-loops, which segregate into two distinct conformations, denoted “up” and “down”, related by a 20° angle [22]. Superposition of the CP subunits in the 80α CP^FA^ reconstruction showed that the E-loops do not fall clearly into two classes, but form a continuum of angles (Figure 7C). In most subunits, the E-loops fall within a 10° range, with the exception of the A subunit, for which the E-loop is in a similar “up” conformation to that in the procapsid. This is presumably a result of the tighter curvature in the pentamer compared to the hexamers.

### 3.3. Structure of the Mature SaPI1 Capsid

**Figure 8:**
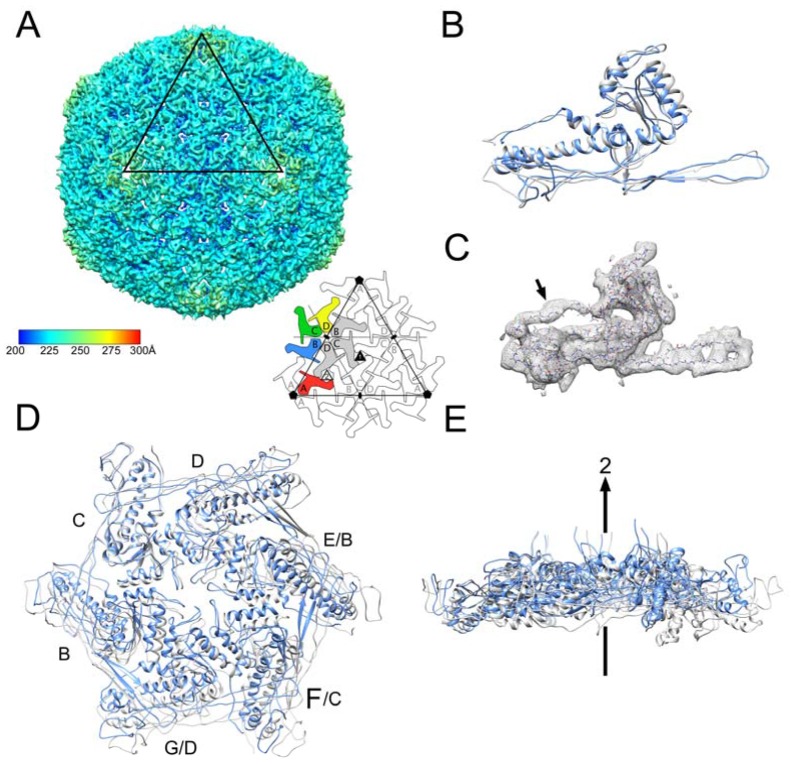
Reconstruction of the SaPI1 capsid. (**A**) Isosurface representation of the SaPI1 reconstruction, colored radially by distance from the capsid center according to the color bar (Å). The triangle representing the icosahedral face is indicated, and corresponds to the schematic diagram to the right. Subunits in one asymmetric unit are colored; symmetry related subunits that make up one hexamer are in gray. (**B**) Superposition of the C subunit in the SaPI1 capsid (gray) on the 80α CP^FA^ capsid (blue). (**C**) Electron density for one CP subunit (mesh), showing density corresponding to the N-arm (arrow). (**D**) Superposition of the SaPI1 hexamer (gray) on the 80α CP^FA^ hexamer (blue), with subunits labeled (A–C for SaPI1; A–G for 80α). Subunit C was used as a reference. (**E**) Same hexamer superposition as in D, rotated by 90°. The twofold axis (2) in SaPI1 is indicated (arrow).

SaPI1 virions were produced by induction of *S. aureus* strain ST65, an 80α Δ*orf44* lysogen that contains SaPI1 *tst::tetM* (Table 1). We previously showed that the deletion of *orf44* had no effect on SaPI1 assembly or viability [21], but results in SaPI1 lysates with no contaminating helper phage. The SaPI1 virions were imaged by cryo-EM in an FEI F20 electron microscope using photographic film (Figure 4B). Most capsids in this case are full. The structure of the DNA-filled SaPI1 mature capsid was determined to 8.4 Å resolution using AUTO3DEM from 6471 particles picked from these images (Figure 4D). The SaPI1 mature capsid displayed the expected *T* = 4 icosahedral architecture comprised of A_5_ pentamers and (BCD)_2_ hexamers located on fivefold and twofold symmetry axes, respectively (Figure 8A). CP subunits A–D derived from the 80α CP^FA^ model were placed into the SaPI1 density by rigid body fitting, followed by real-space refinement in Coot.

As in the procapsids, there is very little difference between CP subunits in 80α and SaPI1 mature capsids. The CP subunits from the 80α CP^FA^ reconstruction could therefore be fitted individually into the SaPI1 density with only minor adjustments of the N-arm and of the α5 and α6 helices in the A-domain (Figure 8B). As noted above, density for the N-arm was apparent, demonstrating that the extended conformation seen in the CP^FA^ capsid was not due to the lack of CP cleavage (Figure 8C). The distribution of CP subunits within hexamers, however, is dramatically different in the 80α and SaPI1 capsids. While hexamers in 80α and SaPI1 procapsids could be superimposed with a root-mean-square deviation (RMSD) of all Cα atoms of 1.9 Å, the RMSD for the mature capsids was 7.0 Å. In the SaPI1 capsid, CP subunits on opposite sides of the hexamer are >11 Å further apart than in 80α, leaving a larger hole in the middle of the hexamer, and the hexamer has a distinct bend of ≈10° relative to 80α (Figure 8D,E). These differences presumably reflect the different environments of the hexamers in the *T* = 7 and *T* = 4 capsids. In the *T* = 7 80α capsids, the hexamers are located on the flat face of the icosahedron, whereas in the *T* = 4 SaPI1 capsids, they sit on the twofold axis at the edges between faces. The pentamers are somewhat more similar between the SaPI1 and 80α capsids (RMSD = 4.1 Å), reflecting less pronounced spreading of CP subunits than in the hexamers.

### 3.4. Comparison of Capsomer Angles

**Figure 9:**
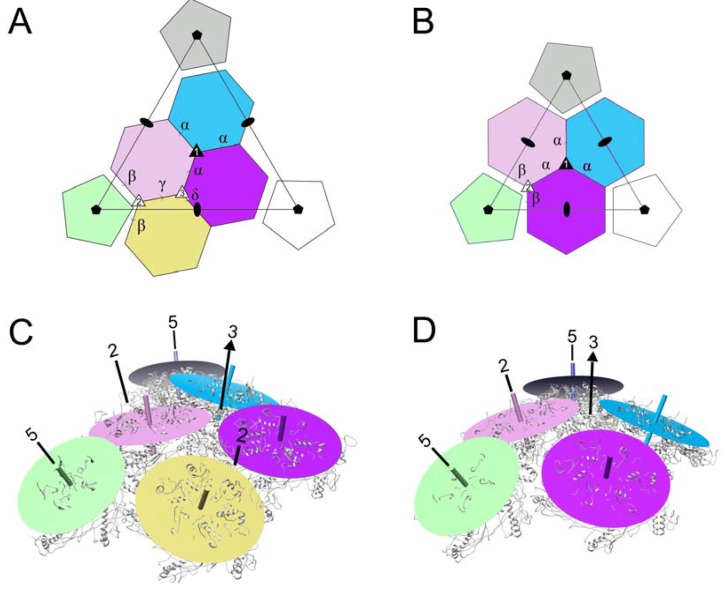
Comparison of capsomer organization in 80α and SaPI1 mature capsids. (**A**,**B**) Schematic diagrams of the *T* = 7 80α (**A**) and *T* = 4 SaPI1 (**B**) lattices, defining the dihedral angles α, β, γ and δ. The angles are listed in Table 3. One icosahedral triangle is shown, and fivefold, threefold and twofold axes are indicated by filled pentagons, triangles and ovals, respectively. The quasi-threefold axes are shown as open triangles. (**C**,**D**) Representation of the 80α (**C**) and SaPI1 (**D**) mature capsids, showing each capsomer as a disc corresponding to a plane drawn between equivalent atoms in the five or six subunits (gray ribbon diagram) that comprise the capsomer, colored as in A and B. Locations of symmetry axes (2, 3, 5) are indicated.

We previously described the dihedral angles between capsomers in the 80α and SaPI1 procapsids, by drawing a plane through equivalent atoms in the hexamers and pentamers and measuring the dihedral angles between these planes [22]. Here, we defined dihedral angles between capsomers in the 80α and SaPI1 mature capsids in the same way (Figure 9A,B). The resulting angles are listed in Table 3. We had expected the dihedral angles between the capsomers related by the icosahedral threefold (α) to be larger (i.e., more planar) in the mature capsid due to the more planar surface on the face of the icosahedron. Surprisingly, the α angles were very similar to those in the procapsid, caused by a “rippling” of the hexamers around the threefold axis (Figure 9C). Along the twofold edge of the icosahedron, the capsomers describe a twisted path, while dihedral angles (β, γ and δ) differ by up to 7.5° from those in the procapsid (Figure 9C; Table 3). This configuration apparently minimizes the perturbation of the threefold interactions upon the transition from a spherical to an icosahedral shell and the distinctly different environments of the icosahedral face and edge in the mature capsids. In the *T* = 4 SaPI1 shell, there is no rippling of hexamers around the threefold axis (Figure 9D), and the dihedral angles α and β are identical to those observed in the procapsid (Table 3). In this case, the distinctly different environment of the hexamer on the twofold axis is accommodated by changes in the hexamer itself (Figure 8C,D).

## 4. Discussion

Capsid expansion accompanied by significant structural changes is a general theme among the tailed bacteriophages (the Caudovirales), first described for phages P22 and λ [41,42]. The expansion process has been well described structurally in HK97, but this is a somewhat atypical case, since this group of phages does not encode a separate scaffolding protein, instead having a scaffolding domain fused to the capsid protein [43]. 80α is a more typical example of a phage with a separate scaffolding protein. We previously showed that 80α SP forms a helix-and-hook motif that makes numerous contacts with the N-arm helix of CP in the procapsid [22]. In this paper, we have shown that the N-arm in the mature capsid is rotated by 81°, accompanied by a straightening of the spine helix (Figure 6D). We hypothesize that this rotation is triggered by the release of the N-arm from SP, thereby coupling the capsid expansion to scaffolding removal. Most likely, the DNA entering the capsid is the direct cause of SP release, perhaps due to electrostatic repulsion between SP and the DNA. Procapsids are considered to exist in a metastable state, so that SP removal and the pressure of the entering DNA would lead to spontaneous expansion [44]. The released SP would have to escape from the capsids, presumably before expansion is complete and the shell is essentially closed. The resulting mature shell is stronger than the procapsid, even though it is thinner. This is apparently accomplished through increased interactions between the A-domains (α5 and α6) and around the threefold axes. The E-loops flex to accommodate this transition, while at the same time tethering the subunits together.

In 80α, both CP and SP undergo cleavage as part of the assembly or maturation process. While cleavage of CP and sometimes SP and other capsid proteins is not unusual in other phages, 80α was the first example of a phage that is cleaved by a host protease [12]. The host protease Prp plays an essential role in normal cellular function, for processing of ribosomal protein L27 during ribosome assembly [12,13]. However, the functional role of the cleavage of CP and SP in 80α is still unclear. Cleavage itself is not the direct trigger for capsid expansion, since procapsids isolated from an 80α infection contain proteins that have already been cleaved [11]. Indeed, cleavage of CP does not seem to be important for assembly or maturation, since 80α mutants making either precleaved (CP*) or uncleavable (CP^FA^) CP both assembled normally and yielded viable phage (Figure 2A,B). SP cleavage *per se* is also not important, since the 80α SP^FA^ mutant is also viable. However, 80α procapsid assembly is absolutely dependent on having an initially intact SP, and the mutant phage (ST247) expressing the mature form of SP, SP*, made no capsids of any kind (Figure 2C). While this might suggest that SP cleavage occurs after procapsid assembly, this would require Prp to access SP and CP inside the procapsids and to escape from the capsids during DNA packaging. It is perhaps more likely that the SP N-terminus is required for proper folding of SP and/or CP, and that in its absence, the misfolded proteins are rapidly degraded. Consistent with this, CP production is impaired in the SP* mutant, similar to a ΔSP deletion [29]. The N-terminal sequence could provide a mechanism to coordinate the proper folding of two proteins by association with a common chaperone. Alternatively, the peptide could serve a regulatory role, similar to the SP of phage P22 [45]. Some other phages have the same N-terminal cleavage motif on non-homologous proteins, such as the CP of phage 80 [9] or the major tail protein of φ13 (Genbank ID: AT017922). The functional relevance of the cleavage is still unclear, but Prp does appear to associate with large self-assembling complexes, such as ribosomes, procapsids and tails.

In both 80α and SaPI1 procapsids, hexamers and pentamers are essentially identical, and the hexamers have a skewed, asymmetric appearance, consistent with their location on a spherical surface [22]. We previously suggested that size redirection is effected by a change in dihedral angles between capsomers mediated by dimers of the CpmB size determination factor. These dihedral angles define a “jaw angle” that determines whether there is room for another hexamer, or if only a pentamer can be incorporated [22].

During 80α procapsid expansion, the skewed, asymmetric hexamers become sixfold symmetric, consistent with the shift from a spherical capsid to one in which capsomers are located on a flat surface (Figure 5). The small, *T* = 4 capsids produced by 80α in the presence of SaPI1 undergo the same expansion process as 80α and a change from skewed to sixfold symmetric hexamers, although in this case the capsomers are located on the twofold edges between icosahedral faces—a rather different environment from that in the *T* = 7 capsids. This difference is accommodated by changes in the hexamer itself, in which the subunits are further apart, resulting in a more open hexamer structure. This arrangement resembles that of the bacteriophage P2/P4 system, in which hexamers in the *T* = 4 P4 capsid are also widened relative to the *T* = 7 capsid of the P2 helper phage [46]. As in the case of 80α/SaPI1, hexamers and pentamers in the P2 and P4 procapsids start out with the same, skewed architecture [47]. Thus, while assembly starts out with the same asymmetric hexamers in both *T* = 7 and *T* = 4 procapsids, the hexamers diverge during maturation to accommodate the different environments in the mature capsids. The final hexamer conformation may be more driven by geometry than by the specific interactions between subunits, reminiscent of the pattern formation model of Marzec and Day [48], which showed that energy minimization of hexamers on a spherical surface led to triangulated lattices that matched those observed in the capsids, and that the final geometry only depended on the size and shape of the capsomers and of the capsid itself. (Their analysis only considered spherical capsids, but the same principles may hold true for the angular mature capsids.) The changes that hexamers (and to a lesser extent, the pentamers) undergo to accommodate not only capsid expansion, but also the different architectures of the *T* = 7 and *T* = 4 shells, emphasize the flexibility of the capsomers to adapt to these different environments, while retaining most of the critical protein-protein interactions needed to stabilize the mature shell.

## Figures and Tables

**Table 1:** List of *S. aureus* strains.

Strain	Genotype/description	Alias	Reference
RN450	Phage cured version of reference strain NCTC 8535	–	[8]
RN4220	Phage cured NCTC 8535 mutated to accept foreign DNA	–	[26]
RN10616	RN4220 (80α)	WT	[27]
JP3569	RN450 (80α ΔCP)	ΔCP	[28]
ST91	RN4220 (80α ΔSP)	ΔSP	[29]
ST65	RN4220 (80α Δ*orf4* 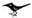 ) SaPI1 *tst::tetM*	SaPI1	[21]
ST209	RN450 (80α CP Δ1-14)	CP*	This work
ST208	RN450 (80α CP::F14A)	CP^FA^	This work
ST247	RN4220 (80α SP Δ1-13)	SP*	This work
ST379	RN4220 (80α SP::F12A,F13A)	SP^FA^	This work

**Table 2:** Representative mutant phage titers, measured as plaque forming units (pfu) per mL lysate.

Strain	Phage	Titer (pfu/mL)
RN10616	80α WT	3.0 × 10^10^
ST208	80α CP^FA^	2.7 × 10^10^
ST209	80α CP*	1.8 × 10^10^
ST379	80α SP^FA^	4.2 × 10^10^
ST247	80α SP*	<10

**Table 3:** List of dihedral angles between capsomers in the 80α and SaPI1 procapsids and mature capsids. For definition of the angles α, β, γ and δ, see Figure 9. Δ refers to the difference in the angle between procapsid and mature capsid.

Angle	80α			SaPI1		
	Procapsid	Mature Capsid	Δ	Procapsid	Mature Capsid	Δ
α	157.1	157.5	0.4	144.0	144.0	0.0
β	152.9	145.8	−7.1	148.3	148.3	0.0
γ	149.0	141.5	−7.5	-	-	-
δ	158.3	163.7	5.4	-	-	-

## Supplementary Materials

The following are available online at www.mdpi.com/1999-4915/9/12/384/s1.
