# Supplementary material for: Cleavage and Structural Transitions during Maturation of Staphylococcus aureus Bacteriophage 80α and SaPI1 Capsids
Source: Viruses. 2017 Dec 16;9(12):384. doi: 10.3390/v9120384 (PMC5744158; doi:10.3390/v9120384)
Supplement: Supplementary file 1 [file viruses-09-00384-s001.zip › Supplementary files/FigS1_gel_withlegend.doc]

**Supplementary Figure S1.** SDS-PAGE showing protein compositions of virions generated by 80α WT and the 80α mutants analyzed in this study: CP^FA^ (ST208), CP* (ST209) and SP^FA^ (ST379). Mutant SP* (ST247), which makes no capsids, was not included. The amount of material loaded (2µl or 10µl) is indicated above the lanes. Bands corresponding to uncleaved CP, CP*, tail protein (TP), portal protein (PP), baseplate proteins (BP) and tail fiber (Fib) are indicated. The 80α WT procapsid and tail fraction (WT pro) is shown for comparison (10µl load). Lane M, marker; molecular weights as indicated (kDa).
