# Supplementary material for: Cleavage and Structural Transitions during Maturation of Staphylococcus aureus Bacteriophage 80α and SaPI1 Capsids
Source: Viruses. 2017 Dec 16;9(12):384. doi: 10.3390/v9120384 (PMC5744158; doi:10.3390/v9120384)
Supplement: Supplementary file 1 [file viruses-09-00384-s001.zip › Supplementary files/FigS2_80a-WT-density_withlegend.doc]

**Supplementary Figure S2.** Density (mesh) for one CP subunit from the previously determined 80α WT mature capsid reconstruction (Spilman et al. 2011, *J. Mol. Biol.* 405, 863-876), sharpened by application of an inverse B factor and rendered at a low cutoff level to show the density corresponding to the N-arm (arrow). The all-alanine molecular model from the 80α CP^FA^ reconstruction is shown placed in the density.
