# Supplementary material for: Cleavage and Structural Transitions during Maturation of Staphylococcus aureus Bacteriophage 80α and SaPI1 Capsids
Source: Viruses. 2017 Dec 16;9(12):384. doi: 10.3390/v9120384 (PMC5744158; doi:10.3390/v9120384)
Supplement: Supplementary file 1 [file viruses-09-00384-s001.zip › Supplementary files/Supplementary Movie Legend.doc]

**Supplementary Movie 1.** Ribbon representation of 80α CP subunit C showing morph between the procapsid and the mature capsid. In the procapsid, the N-arm is shown in pink, helix α2 and part of the spine helix (α3) up to residue P132 are shown in light green, and the SP is shown in purple. At the mature capsid endpoint, the pink and light green colors darken to red and green.
