# Supplementary material for: Cleavage and Structural Transitions during Maturation of Staphylococcus aureus Bacteriophage 80α and SaPI1 Capsids
Source: Viruses. 2017 Dec 16;9(12):384. doi: 10.3390/v9120384 (PMC5744158; doi:10.3390/v9120384)
Supplement: Supplementary file 1 [file viruses-09-00384-s001.zip › Supplementary files/TableS1_plasmids.doc]

**Table S1** Plasmids.

| **Plasmid** | **Description** | **Reference** |
| --- | --- | --- |
| pMAD | *E. coli* – *S. aureus* shuttle vector for allelic exchange | [24] |
| pEW13 | pMAD with 80α SP Δ1-13 | This work |
| pJMB2 | pMAD with 80α CP Δ1-14 | This work |
| pLAK6 | pMAD with 80α SP::F12A, F13A | This work |
| pMED2 | pMAD with 80α CP::F14A | This work |
