# Supplementary material for: Cleavage and Structural Transitions during Maturation of Staphylococcus aureus Bacteriophage 80α and SaPI1 Capsids
Source: Viruses. 2017 Dec 16;9(12):384. doi: 10.3390/v9120384 (PMC5744158; doi:10.3390/v9120384)
Supplement: Supplementary file 1 [file viruses-09-00384-s001.zip › Supplementary files/TableS2_oligos.doc]

**Table S2**. Oligonucleotides used in this study.

| **Plasmid** | **Oligonucleotides** | **Sequence (5’ - 3’)** |
| --- | --- | --- |
| **pEW13** |  |  |
|  | EAW5 (NcoI) | CGATGCATG**CCATGG***TGGTCGAAAACAAGGACTTTAGCGATAG |
|  | EAW56 | ATCTGATTGGTCTGC*CATGAGATATACCTCCATTTATAGTCTGTCG |
|  | EAW57 | GCAGACCAATCAGAT*GATCCGGACGAACCAGG |
|  | EAW8 (BamHI) | GGCGATATC**GGATCC***TTCCAATGATTTCGGGCATGTTAC |
| **pJMB2** |  |  |
|  | GC47IF1 (NcoI) | TCGATGCATG**CCATGG***AGACCAATCAGATGATCCGGACGAA |
|  | GC47*R | CATTTAAATGCCTCC*GTTAATTTTTAA |
|  | GC47*F | GGAGGCATTTAAATG*GCGAGTAACAA |
|  | GC47IF2 (BamHI) | GGGCGATATC**GGATCC***GGGTTAATATTGCATGGTATCGCATCA |
| **pLAK6** |  |  |
|  | EAW5 | CGATGCATG**CCATGG***TGGTCGAAAACAAGGACTTTAGCGATAG |
|  | EAW175 | TGC(TGCTGC)TTGCAA*ATTAAACTTAAGTTTATTTTCTTCCATGAGATATACC |
|  | EAW176 | TTGCAA(GCAGCA)GCA*GACCAATCAGATGATCCGGAC |
|  | EAW8 | GGCGATATC**GGATCC***TTCCAATGATTTCGGGCATGTTAC |
| **pMED2** |  |  |
|  | GC47-IF1 (NcoI) | TCGATGCATG**CCATGG***AGACCAATCAGATGATCCGGACGAA |
|  | GC47-FA2 | ACATTGTTACTCGCA*(GCA)TGTTGCAAATTTAATTTTAATTTTTGTGTTTGTTC |
|  | GC47-FA3 | TGCGAGTAACAATGT*TAAACCGCAAGT |
|  | GC47-IF2 (BamHI) | GGGCGATATC**GGATCC***GGGTTAATATTGCATGGTATCGCATCA |

The annealing sequence for amplification is underlined and the 5’ 15-16 bp overhang required for the In-Fusion reaction is separated from the rest of the primer by an asterisk (*). Restriction sites used to linearize the plasmid prior to assembly are noted next to the oligo name, and shown in bold in the sequence when they were reconstituted in the finished assembly. Introduced Ala codon substitutions are shown in parentheses.
